# Attitudes Toward Family-Based Treatment Impact Therapists’ Intent to Change Their Therapeutic Practice for Adolescent Anorexia Nervosa

**DOI:** 10.3389/fpsyt.2020.00305

**Published:** 2020-04-23

**Authors:** Erin C. Accurso, Daniel Le Grange, Andrea K. Graham

**Affiliations:** ^1^ Department of Psychiatry, UCSF Weill Institute for Neurosciences, University of California, San Francisco, San Francisco, CA, United States; ^2^ Department of Psychiatry & Behavioral Neuroscience, The University of Chicago, Chicago, IL, United States; ^3^ Department of Medical Social Sciences, Northwestern University Feinberg School of Medicine, Chicago, IL, United States

**Keywords:** family-based treatment, eating disorders, children and adolescents, dissemination and implementation, clinician attitudes

## Abstract

Community-based clinicians who treat patients with eating disorders rarely use empirically-supported treatments, and research demonstrates that clinicians make significant modifications when implementing family-based treatment (FBT) for anorexia nervosa. This study examined clinician attitudes toward FBT and explored the extent to which attitudes predicted intent to shift practices following training in FBT. Clinicians (*N* = 129) completed a standardized training in FBT for AN, either a two-day introductory training (*n* = 99) or a one-day “advanced” training (*n* = 30). Linear regressions were used to examine the association between therapists’ attitudes toward FBT and their intent to use strategies consistent with FBT in the future, adjusting for pre-training use of strategies. Providers reported very positive attitudes toward evidence-based practices in general and moderately positive attitudes toward FBT. There were no significant differences between “novice” and “advanced” providers on attitudes toward evidence-based practices or FBT (*p*s > .10). For the subset of providers attending their first training in FBT, more positive attitudes toward FBT significantly predicted greater intent to use FBT-consistent strategies (*p* = .004), and more positive attitudes toward evidence-based practice significantly predicted lesser intent to use FBT-inconsistent strategies (*p* = .009). This study suggests that both general attitudes toward evidence-based practice and specific attitudes toward FBT may impact implementation. Future research might examine whether a brief intervention to improve attitudes toward FBT might increase the likelihood of seeking expert consultation post-training.

## Introduction

Anorexia nervosa (AN) has severe medical and psychological consequences (1–4) and the highest mortality rate of any psychiatric disorder (5). Research supports family-based treatment (FBT) as an efficacious treatment for youth with AN (6), and initial studies generally support the effectiveness of FBT when disseminated to teaching hospitals (7, 8). However, community-based clinicians who treat patients with eating disorders rarely use empirically-supported treatments (9, 10), and the use of FBT in “usual care” is no exception. Clinicians who do utilize FBT often make significant modifications in its implementation (11) that may impact its effectiveness. One study that coded for fidelity found that almost three-quarters of clinicians had at least considerable fidelity initially, but this decreased to about half of clinicians in the second and third phases of FBT (7). Therefore, understanding factors related to adoption and implementation is critical.

Common provider-level barriers to the use of empirically-supported treatments are perceptions that these treatments are too rigid or inadequate for the complexity of cases seen in their practice (10, 12–14). Understanding therapist attitudes and beliefs about evidence-based treatments may help to inform dissemination and implementation efforts. Preliminary research shows that patients view FBT as effective and acceptable (8, 15). Preliminary data also suggest that community-based clinicians may hold negative beliefs about FBT that decrease over the course of supervision and implementation. However, a better understanding of these beliefs may be helpful to more fully appreciate what may prevent clinicians from seeking additional supervision and implementing FBT (16).

Therefore, the main aims of this study were to: 1) examine treatment provider attitudes toward FBT (in order to identify potential barriers to implementation), and 2) test whether treatment provider attitudes predict intent to use FBT-consistent strategies with greater frequency in the future.

## Methods

Clinician participants (*N* = 129) enrolled through a training institute in eating disorders for a fee and completed training in FBT for AN in New Jersey or Illinois between 2012 and 2013. Most clinicians attended one of three standardized two-day introductory training (*n* = 99); the remainder (*n* = 30) attended an “advanced” training; all trainees participated in this study. The introductory training included a review of the research literature related to treatment of adolescent AN, an overview of the main tenets of FBT and the treatment frame, an overview of each phase and specific techniques at each phase/key sessions of FBT, role-plays, common challenges in FBT, and opportunities for questions/discussion. The advanced training focused on complex case presentations and complicating factors in implementing FBT, including role-plays to illustrate advanced skills. Pre-training, clinicians provided information about their clinical experience and reported on their use of a range of different treatment techniques for adolescents with AN using the Therapeutic Strategy Checklist for Adolescent Anorexia Nervosa. Post-training (last activity of the day on the last day of training), clinicians reported on their intent to use the same set of treatment techniques in the future and completed the Family-Based Treatment Attitude Scale. The institutional review board at the University of Chicago approved all protocols.

### Measures

#### Therapeutic Strategy Checklist for Adolescent Anorexia Nervosa (TSC-AN)

The TSC-AN, which developed for the current study, includes a list of 25 treatment techniques that may be used in the treatment of adolescents with AN (e.g., provide education about the mortality and morbidity associated with AN, externalize the disorder to reduce blame of the parents and the adolescent, weigh adolescent at every session) (see **Appendix A** in the **Supplemental Material**). A total of 40 items were initially developed to attempt to capture a range of interventions that might be used in the treatment of adolescent AN, including key FBT interventions. In collaboration with one of the FBT treatment developers and five other experts in adolescent eating disorders, the list was reduced to the 25 items that comprise this measure, incorporating edits to clarify and simplify wording. Pre-training, clinicians reported on the frequency with which they *currently use each strategy* with adolescent AN cases in the first 2–3 months of treatment from 0 (never) to 4 (almost always or always). Some of the treatment components are consistent with FBT (*n* = 12) and other elements are inconsistent with FBT (*n* = 8); other treatment elements were neither consistent nor incompatible with FBT (*n* = 5). The FBT-consistent strategies (range: [0, 48]) and FBT-inconsistent strategies subscale scores (range: [0, 32]) were used for this study. Post-training, clinicians completed a parallel version of the same measure in which they reported on the frequency with which they *intend to use each strategy in the future*. The FBT-consistent strategies demonstrated good reliability with a Cronbach’s alpha of.83. FBT-inconsistent strategy items would be expected to demonstrate poor interdependence due to their heterogeneity in theoretical underpinnings—in addition to a small number of items—consistent with a Cronbach’s alpha of .66.

#### Evidence-Based Practice Attitude Scale (EBPAS)

The EBPAS (17) is a 15-item measure that assesses therapists’ attitudes toward the adoption of manualized evidence-based treatments, with higher scores indicating more positive attitudes toward evidence-based practices [range: 0, 4]. The EBPAS does not ask about specific practices. Its total score indicates global attitudes toward adoption of evidence-based practices, with good reliability and predictive validity (18, 19) and a Cronbach’s alpha of.83 in this sample.

#### Family-Based Treatment Attitude Scale (FBT-AS)

The FBT-AS, which was developed for the current study, is a 20-item measure that assesses clinician attitudes toward FBT for AN (see **Appendix B** in the **Supplemental Material**). The initial 30 items were developed to capture four domains that would impact implementation, including perceived relevance and clinical appropriateness of FBT (e.g., *FBT is relevant to the needs of the adolescent AN cases that present to my practice*), treatment credibility (e.g., *FBT can be an effective treatment for my adolescent clients with AN*), implementation confidence (e.g., *I feel confident that I could successfully implement FBT with further supervision*), and feasibility (e.g., *My agency will not provide me with the resources and/or time necessary to be competent in FBT*). After review by six experts in adolescent eating disorders (including one of the FBT treatment developers), the list was reduced to the 20 items that comprise this measure, incorporating edits to clarify and simplify wording. Items are rated from −2 (strongly disagree) to 2 (strongly agree), with higher scores indicating more favorable attitudes toward FBT [range: −40, 40]. The scale demonstrated good internal reliability in the current sample (Cronbach’s alpha = .84), as well as a small positive correlation with the EBPAS total (*r* = .33, *p* = .012).

### Analyses

Linear regressions were used to examine the association between therapists’ attitudes toward FBT and their intent to use strategies consistent with FBT in the future, adjusting for pre-training use of strategies. Regressions including all three independent variables were adequately powered (.81) to detect a medium effect size (Cohen’s d = 0.3) (20).

## Results

Therapist participants were primarily women (*n* = 115, 89.1%) with a mean age of 37.6 years (*SD* = 9.3) who had been practicing therapy for 9.5 years (*SD* = 8.4) on average. The majority of clinicians worked in settings that offered traditional outpatient treatment (*n* = 108, 86.4%). In addition, their settings offered intensive outpatient and/or partial hospitalization programs (*n* = 42, 33.6%), residential treatment (*n* = 13, 10.4%), and inpatient treatment (*n* = 38, 30.4%). Eating disorders cases comprised approximately two-thirds of cases on their caseload (*M* = 63.8, *SD* = 37.7), and about three-fifths of their cases were 18 years of age or younger (*M* = 57.1, *SD* = 30.8). Given that there were no significant differences in demographic characteristics between “novice” providers who were attending an introductory training in FBT (*n* = 99) and “advanced” providers who were attending an advanced training in FBT (*n* = 30) (*p*s > .10), the data are presented in aggregate in **Table 1**. At pre-training, three-fifths (59.1%, *n* = 74) of all providers reported that they would recommend a higher level of care than would be typically warranted for a medically-stable adolescent with AN, including intensive outpatient (34.1%, *n* = 42), partial hospitalization (15.4%, *n* = 19), and residential or inpatient (10.6%, *n* = 13). Prior training/expertise in FBT was relatively low, with 9.6% (n = 12) of the total sample reporting training in FBT to a “great” or “very great” extent. There were no significant differences between “novice” and “advanced” providers on level of care recommendations or prior training in FBT (*p*s > .10).

**Table 1:** Characteristics of therapists in the full sample (*N* = 129).

Therapist Characteristics	*M* (*SD*) or %
Gender (female)	89.9%
Age (yrs)	37.8 (9.5)
Therapy experience (yrs)	9.8 (8.7)
*Discipline*	
Psychology	40.8%
Social work	33.7%
Other	25.5%
*Primary theoretical orientation*	
Cognitive behavioral or behavioral	44.8%
Eclectic	17.7%
Family systems	6.3%
Psychodynamic	11.5%
Other	19.7%
*Primary employment setting*	
Private practice	35.7%
Hospital	27.6%
For-profit agency	13.3%
Community mental health clinic	11.2%
University-affiliated clinic	9.2%
Other	3.1%
*Levels of care offered at employment setting*	
Outpatient treatment	87.5%
Intensive outpatient and/or partial hospitalization	36.5%
Residential	11.5%
Inpatient	30.2%
Caseload comprised of eating disorders patients (%)	65.2 (37.1)
Caseload comprised of 18 and under patients (%)	59.9 (33.8)

### Current Use of Treatment Strategies

**Figure 1:**
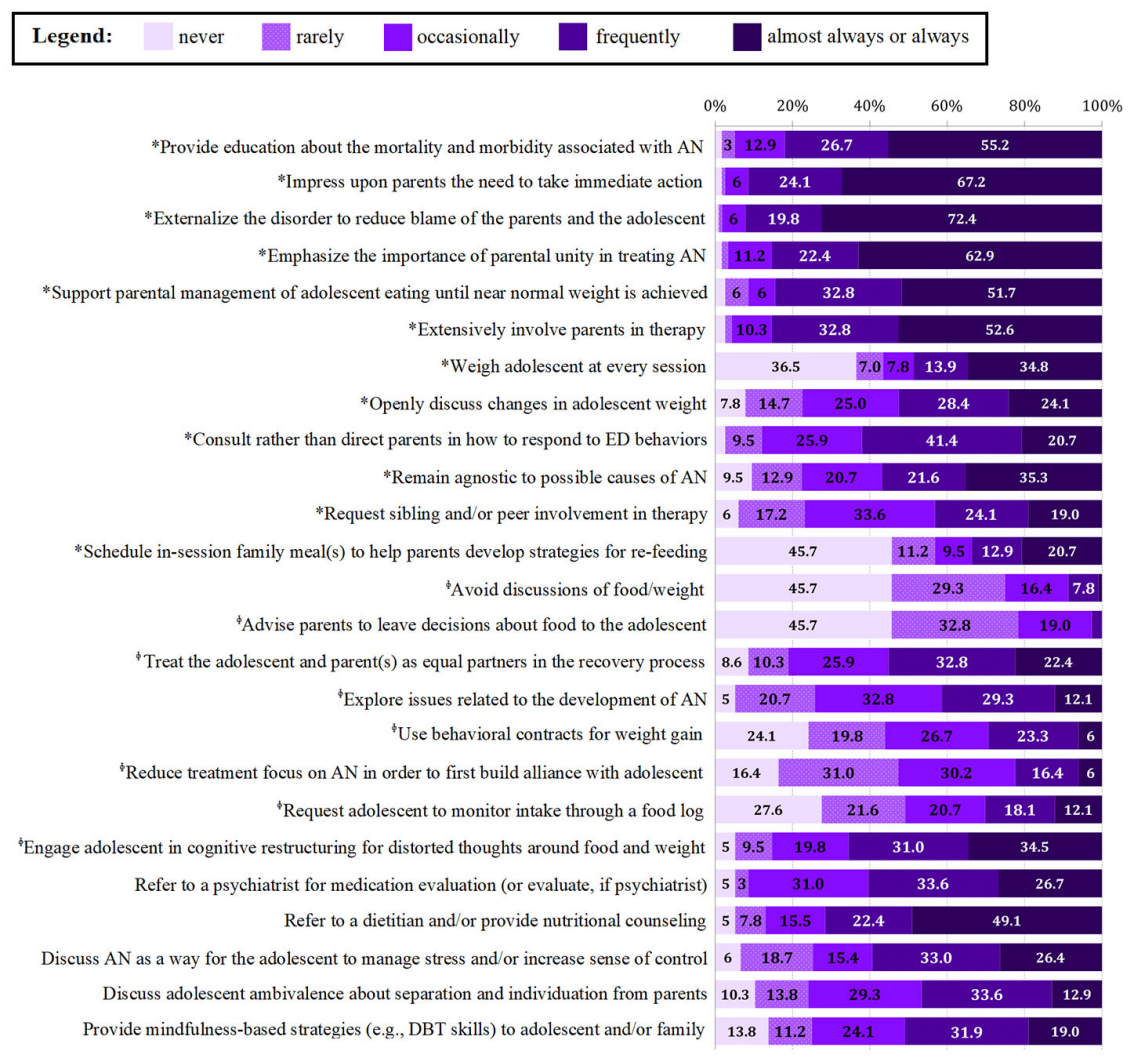
Pre-training therapeutic strategy use in the first three months of treatment for adolescent anorexia nervosa (AN). Note: *Family-based treatment (FBT)-consistent strategies; ϕ FBT-inconsistent strategies; values less than 7% are rounded down to the nearest whole number, and those less than 3% are not noted numerically due to space.

Several treatment strategies consistent with FBT were *frequently* to *always* utilized by most clinicians, including providing education about mortality and morbidity (82.4%, *n* = 75), impressing upon the parents the need to take immediate action (92.3%, *n* = 84), externalizing the eating disorder (92.3%, *n* = 84), and supporting parental management of eating (85.7%, *n* = 78). However, only about half assessed weight at each session (47.8%, *n* = 43) or openly discussed changes in weight (55.0%, *n* = 50). Across the 25 treatment strategies assessed, there were no significant differences in frequency of use between providers (*p*s > .10) with the exception that “novice” providers were significantly more likely to use behavioral contracts for weight gain than “advanced” providers (*t* = −3.38, *p* = .001; *M* = 1.87, *SD* = 1.21 v. *M* = 0.96, *SD* = 1.10). **Figure 1** provides the reported frequencies of use for each treatment strategy at pre-training across the full sample. Current use of FBT-consistent strategies and FBT-inconsistent strategies did not significantly differ between groups (*p*s > .10).

### Attitudes Toward and Intent to Use FBT

Prior to training, providers reported very positive attitudes toward evidence-based practices in general (*M* = 3.08, *SD* = 0.50). Following training, providers also had moderately positive attitudes toward FBT specifically (*M* = 20.30, *SD* = 9.23). There were no significant differences between “novice” and “advanced” providers on attitudes toward evidence-based practices or FBT (*p*s > .10). Intent to use FBT-consistent strategies post-training (*M* = 44.61, *SD* = 11.72) was significantly greater than use at pre-training (*M* = 34.56, *SD* = 8.12, *t* = −7.84, *p* < .001). Intent to use FBT-inconsistent strategies post-training (*M* = 8.57, *SD* = 4.33) was significantly less than use at pre-training (*M* = 11.55, *SD* = 4.33, *t* = −5.85, *p* < .001). Post-training intent to use FBT-inconsistent strategies in the future did not significantly differ between groups (*p* > .10), but “advanced” providers reported post-training intent to use FBT-consistent strategies more frequently in the future than “novice” providers (*t* = 3.69, *p* < .001; *M* = 52.05, *SD* = 21.60 v. *M* = 42.51, *SD* = 5.16).

For the subset of providers attending their first training in FBT, we examined whether providers’ baseline attitudes toward evidence-based practices or post-training attitudes toward FBT influenced their reported intent to increase use of FBT-consistent strategies or decrease use of FBT-inconsistent strategies in the future. More positive attitudes toward FBT predicted greater intent to use FBT-consistent strategies in the future (*B* = 0.224, *SE* = .055, *t* = 4.042, *p* < .001, adjusted R^2^ = .116), adjusting for current strategy use (overall model: *F* = 28.253, *p* < .001, adjusted R^2^ = .408). More positive baseline attitudes toward evidence-based practices also predicted greater intent to use FBT-consistent strategies in the future (*B* = 2.826, *SE* = 1.252, *t* = 2.257, *p* = .028, adjusted R^2^ = .046), adjusting for current strategy use (overall model: *F* = 15.265, *p* < .001, adjusted R^2^ = .338). When both variables were entered into a model simultaneously, the overall model predicting intent to use FBT-consistent strategies remained significant (*F* = 14.821, *p* < .001, adjusted R^2^ = .425). Adjusting for current strategy use, more positive attitudes toward FBT significantly predicted greater intent to use FBT-consistent strategies (*B* = 0.201, *SE* = 0.066, *t* = 3.043, *p* = .004) whereas more positive attitudes toward evidence-based practice at baseline did not (*B* = 2.129, *SE* = 1.188, *t* = 1.792, *p* = .08).

More positive attitudes toward FBT predicted lesser intent to use FBT-inconsistent strategies in the future (*B* = −0.203, *SE* = 0.054, *t* = −3.792, *p* < .001, adjusted R^2^ = .113), adjusting for current strategy use (overall model: *F* = 12.591, *p* < .001, adjusted R^2^ = .227). Similarly, more positive baseline attitudes toward evidence-based practices predicted lesser intent to use FBT-inconsistent strategies (*B* = −0.203, *SE* = 0.054, *t* = −3.786, *p* < .001, adjusted R^2^ = .172), adjusting for current strategy use (overall model: *F* = 10.776, *p* < .001, adjusted R^2^ = .266). When both variables were entered into a model simultaneously, the overall model predicting intent to use FBT-inconsistent strategies remained significant (*F* = 8.389, *p* < .001, adjusted R^2^ = .291). Adjusting for current strategy use, more positive baseline attitudes toward evidence-based practice significantly predicted lesser intent to use FBT-inconsistent strategies (*B* = −2.421, *SE* = 1.261, *t* = −2.714, *p* = .009) whereas more positive post-training attitudes toward FBT did not (*B* = −0.149, *SE* = 0.088, *t* = −1.688, *p* = .10).

## Discussion

This study found that providers who attend introductory or advanced workshops in FBT already report using various techniques that are consistent with FBT, but also multiple techniques that are incompatible with FBT, some of which would require a significant shift in order to implement FBT. Interestingly, there were generally no pre-training differences between “novice” or “advanced” providers in their overall use of strategies. This is not perhaps surprising given that brief workshops increase provider knowledge but are insufficient to change provider behavior (21). It is also not entirely unexpected that a large percentage of providers generally recommend a higher level of care than traditional outpatient treatment since nearly half of providers worked in a setting that provided intensive outpatient, partial hospitalization, and/or residential programs.

Providers intended to use practices consistent with FBT more frequently and those inconsistent with FBT less frequently following training, although the intended shift in practice was far from implementing FBT as a manualized treatment. On average, providers reported very positive attitudes toward evidence-based practice generally and moderately positive attitudes toward FBT specifically despite several barriers, including the focus on higher levels of care in many of their employment settings. For providers exposed to their first training in FBT, both attitudes toward evidence-based practice and attitudes toward FBT mattered for implementation, but for different targets. More positive attitudes about FBT specifically predicted greater intended early uptake of FBT-consistent therapeutic techniques (e.g., openly discussing changes in weight, support parental management of eating), while attitudes about evidence-based practices generally predicted greater intent to reduce use of therapeutic techniques that would interfere with FBT implementation early in treatment (e.g., reducing treatment focus on AN to build an alliance with the adolescent, exploring issues related to the development of AN).

Given evidence that workshop attendance may improve attitudes toward evidence-based practice (22), it may also be worthwhile to identify and target negative attitudes about FBT specifically. Improved attitudes toward FBT may increase the likelihood of providers seeking expert consultation and supervision following the training, which does predict increased clinical skill and adoption of the intervention (21, 23). Providers need to actively choose to initiate supervision or expert consultation, so a brief intervention that could positively impact early attitudes about FBT and increase the likelihood of seeking consultation post-training might in turn increase the odds of implementation “intent” becoming “action.” Further, supervision could provide an additional venue for improving general attitudes toward evidence-based practice. Indeed, prior research indicates that participation in group consultation calls with an expert may improve attitudes toward evidence-based practice (24) and that more open attitudes toward evidence-based practice are associated with greater use of evidence-based treatment techniques (25) and more fidelity-consistent adaptations (26).

Nevertheless, setting and organizational factors impacting implementation would also need to be considered given their link to evidence-based practice use (27) and adherence (25). In addition to not examining organizational factors, this study has several additional limitations, including the relatively small sample that self-selected to attend an FBT training, the use of newly-developed measures with limited psychometric data on their reliability and validity, and short-term outcomes (28) that included reported use of therapeutic practices and reported intent to use therapeutic practices immediately post-training, which may be different than actual behavior or intent even just a couple of weeks following the training. Further, intended shifts in practice were confined to the first two to three months of treatment, which generally constitutes just over half of treatment sessions, primarily focused on phase I of FBT. Despite a biased sample given their proactive choice to attend an FBT training, provider attitudes toward FBT were only moderately positive, and there were no differences demonstrated between the “novice” and “advanced” groups.

Findings suggest that providers use some strategies consistent with FBT prior to formal training in FBT, and that they planned to significantly change their practice following training to be more in line with FBT principles. However, their intended changes were perhaps far from what would be required to implement FBT. Given that provider attitudes about FBT and evidence-based practice predicted greater intended shifts in practice, brief interventions intended to improve attitudes may have a role in increasing adoption. Future research may target negative attitudes toward FBT to determine whether these can be diminished or reversed, and whether doing so increases FBT uptake.

## Data Availability Statement

The raw data supporting the conclusions of this article will be made available by the authors, without undue reservation, to any qualified researcher.

## Ethics Statement

The studies involving human participants were reviewed and approved by University of Chicago. The patients/participants provided their verbal informed consent to participate in this study.

## Author Contributions

EA developed the two new measures (TSC-AN and FBT-AS), designed the study, collected data, developed the data analytic plan, analyzed and interpreted the data, and drafted the manuscript. DL provided feedback on items in both the TSC-AN and FBT-AS. All authors aided in the interpretation of the data, edited the manuscript, and approved the final version of the manuscript.

## Funding

The authors are supported by the National Institutes of Mental Health [Accurso: K23 MH120347] and the National Institute of Diabetes and Digestive and Kidney Diseases [Graham: K01 DK116925].

## Conflict of Interest

DL receives royalties from Guilford Press and Routledge for books on FBT and is co-director of the Training Institute for Child and Adolescent Eating Disorders, LLC, which trains professionals in FBT and other treatment modalities.

The remaining authors declare that the research was conducted in the absence of any commercial or financial relationships that could be construed as a potential conflict of interest.

## Supplementary Material

Appendix A Therapeutic strategy checklist for adolescent anorexia nervosa (TSC-AN).

Appendix B Family-based treatment attitude scale (FBT-AS).
