# Supplementary material for: Attitudes Toward Family-Based Treatment Impact Therapists’ Intent to Change Their Therapeutic Practice for Adolescent Anorexia Nervosa
Source: Front Psychiatry. 2020 Apr 23;11:305. doi: 10.3389/fpsyt.2020.00305 (PMC7192208; doi:10.3389/fpsyt.2020.00305)
Supplement: Appendix A — Therapeutic strategy checklist for adolescent anorexia nervosa (TSC-AN). [file DataSheet_1.docx]

Appendix A. Therapeutic Strategy Checklist for Adolescent Anorexia Nervosa (TSC-AN)

Following the initial assessment, how frequently do you *currently use* the following strategies with adolescent AN cases early in therapy (i.e., within the first 2-3 months)?

|  |  |  | 0 | 1 | 2 | 3 | 4 |
| --- | --- | --- | --- | --- | --- | --- | --- |
|  |  |  | Never | Rarely | Occasionally | Frequently | Almost Always or Always |
| 1. |  | Provide education about the mortality and morbidity associated with AN | ○ | ○ | ○ | ○ | ○ |
| 2. |  | Reduce treatment focus on AN in order to first build alliance with adolescent | ○ | ○ | ○ | ○ | ○ |
| 3. |  | Use behavioral contracts for weight gain | ○ | ○ | ○ | ○ | ○ |
| 4. |  | Impress upon parents the need to take immediate action | ○ | ○ | ○ | ○ | ○ |
| 5. |  | Externalize the disorder to reduce blame of the parents and the adolescent | ○ | ○ | ○ | ○ | ○ |
| 6. |  | Engage adolescent in cognitive restructuring for distorted thoughts around food and weight | ○ | ○ | ○ | ○ | ○ |
| 7. |  | Support parental management of adolescent eating until near normal weight is achieved | ○ | ○ | ○ | ○ | ○ |
| 8. |  | Schedule in-session family meal(s) to help parents develop strategies for re-feeding | ○ | ○ | ○ | ○ | ○ |
| 9. |  | Remain agnostic to possible causes of AN | ○ | ○ | ○ | ○ | ○ |
| 10. |  | Consult rather than direct parents in how to respond to ED behaviors | ○ | ○ | ○ | ○ | ○ |
| 11. |  | Refer to a dietician and/or provide nutritional counseling (e.g., meal plan) | ○ | ○ | ○ | ○ | ○ |
| 12. |  | Treat the adolescent and parent(s) as equal partners in the recovery process | ○ | ○ | ○ | ○ | ○ |
| 13. |  | Advise parents to leave decisions about food to the adolescent | ○ | ○ | ○ | ○ | ○ |
| 14. |  | Request adolescent to monitor intake through a food log | ○ | ○ | ○ | ○ | ○ |
| 15. |  | Weigh adolescent at every session | ○ | ○ | ○ | ○ | ○ |
| 16. |  | Openly discuss changes in adolescent weight | ○ | ○ | ○ | ○ | ○ |
|  |  |  | 0 | 1 | 2 | 3 | 4 |
|  |  |  | Never | Rarely | Occasionally | Frequently | Almost Always or Always |
| 17. |  | Provide mindfulness-based treatment (e.g., DBT) to adolescent and/or family | ○ | ○ | ○ | ○ | ○ |
| 18. |  | Extensively involve parents in therapy for AN | ○ | ○ | ○ | ○ | ○ |
| 19. |  | Explore issues related to the development of AN | ○ | ○ | ○ | ○ | ○ |
| 20. |  | Refer to a psychiatrist for medication evaluation (or evaluate for medication, if psychiatrist) | ○ | ○ | ○ | ○ | ○ |
| 21. |  | Avoid discussions of food/weight | ○ | ○ | ○ | ○ | ○ |
| 22. |  | Emphasize the importance of parental unity in treating AN | ○ | ○ | ○ | ○ | ○ |
| 23. |  | Discuss adolescent ambivalence about separation and individuation from parents | ○ | ○ | ○ | ○ | ○ |
| 24. |  | Request sibling and/or peer involvement in therapy | ○ | ○ | ○ | ○ | ○ |
| 25. |  | Discuss AN as a way for the adolescent to manage stress and/or increase sense of control | ○ | ○ | ○ | ○ | ○ |
|  |  |  |  |  |  |  |  |

Appendix B. Family-Based Treatment Attitude Scale (FBT-AS)

The following questions ask about your attitudes and beliefs about family-based treatment (FBT) for adolescent anorexia nervosa (AN). Please fill in the circle indicating the extent to which you agree or disagree with each item. There are *no right or wrong answers*.

|  | |  | |  | | -2 | | -1 | | 0 | | 1 | | 2 | |
| --- | --- | --- | --- | --- | --- | --- | --- | --- | --- | --- | --- | --- | --- | --- | --- |
|  | |  | |  | | Strongly Disagree | | Disagree | | Neither Agree nor Disagree | | Agree | | Strongly Agree | |
| 1. | |  | | FBT is relevant to the needs of the adolescent AN cases that present to my practice. | | ○ | | ○ | | ○ | | ○ | | ○ | |
| 2. | |  | | FBT can be an effective treatment for my adolescent clients with AN. | | ○ | | ○ | | ○ | | ○ | | ○ | |
| 3. | |  | | FBT would require expensive and/or time-consuming supervision for me to become competent. | | ○ | | ○ | | ○ | | ○ | | ○ | |
| 4. | |  | | FBT makes therapists too much like technicians and too little like skilled, empathic therapists. | | ○ | | ○ | | ○ | | ○ | | ○ | |
| 5. | |  | | I know enough about nutrition to help parents refeed their underweight child. | | ○ | | ○ | | ○ | | ○ | | ○ | |
| 6. | |  | | FBT overemphasizes therapeutic techniques over therapeutic alliance. | | ○ | | ○ | | ○ | | ○ | | ○ | |
| 7. | |  | | FBT can work as well as—if not better—than my current treatment methods for adolescent AN. | | ○ | | ○ | | ○ | | ○ | | ○ | |
| 8. | |  | | FBT appropriately attends to individual differences in adolescents with AN. | | ○ | | ○ | | ○ | | ○ | | ○ | |
| 9. | |  | | I feel confident that I can successfully implement FBT *without* further supervision. | | ○ | | ○ | | ○ | | ○ | | ○ | |
| 10. | |  | | I feel confident that I could successfully implement FBT *with* further supervision. | | ○ | | ○ | | ○ | | ○ | | ○ | |
| 11. | |  | | FBT focuses too much weight restoration and not enough on other problems (e.g., anxiety). | | ○ | | ○ | | ○ | | ○ | | ○ | |
| 12. | |  | | I may feel unfulfilled or bored as a therapist if I were to administer FBT. | | ○ | | ○ | | ○ | | ○ | | ○ | |
|  |  | |  | | -2 | | -1 | | 0 | | 1 | | 2 | |  |
|  |  | |  | | Strongly Disagree | | Disagree | | Neither Agree nor Disagree | | Agree | | Strongly Agree | |  |
| 13. |  | | FBT does not fit with the treatment philosophy at my agency. | | ○ | | ○ | | ○ | | ○ | | ○ | |  |
| 14. |  | | My agency will not provide me with the resources and/or time necessary to be competent in FBT. | | ○ | | ○ | | ○ | | ○ | | ○ | |  |
| 15. |  | | The nature of FBT would prevent me from being authentic and genuine with families. | | ○ | | ○ | | ○ | | ○ | | ○ | |  |
| 16. |  | | I feel concerned about being the primary treatment provider in the care of an underweight adolescent. | | ○ | | ○ | | ○ | | ○ | | ○ | |  |
| 17. |  | | I would likely feel uncreative or constrained while administering FBT. | | ○ | | ○ | | ○ | | ○ | | ○ | |  |
| 18. |  | | My clients will not be able and/or willing to participate in FBT for adolescent AN. | | ○ | | ○ | | ○ | | ○ | | ○ | |  |
| 19. |  | | FBT ignores important individual adolescent and family differences. | | ○ | | ○ | | ○ | | ○ | | ○ | |  |
| 20. |  | | FBT adequately addresses diagnostic comorbidity and/or multiple problems areas. | | ○ | | ○ | | ○ | | ○ | | ○ | |  |
| 21. |  | | FBT would interfere with forming a strong therapeutic alliance with the adolescent. | | ○ | | ○ | | ○ | | ○ | | ○ | |  |
| 22. |  | | FBT would interfere with forming a strong therapeutic alliance with the parent(s). | | ○ | | ○ | | ○ | | ○ | | ○ | |  |
| 23. |  | | FBT provides adolescents with an appropriate level of independence. | | ○ | | ○ | | ○ | | ○ | | ○ | |  |
| 24. |  | | I am concerned that widespread use of FBT will retard clinical innovation in eating disorders. | | ○ | | ○ | | ○ | | ○ | | ○ | |  |
